# Aprimorando a Avaliação do Edema Pulmonar na Cardiopatia Congênita Pediátrica: O Papel da Ultrassonografia Pulmonar

**DOI:** 10.36660/abc.20250322

**Published:** 2026-04-14

**Authors:** Yasemin Nuran Donmez, Derya Bako, Safak Alpat, Dilek Giray, Serdar Epcacan

**Affiliations:** 1 Van Training and Research Hospital Van Turquia Van Training and Research Hospital, Van – Turquia

**Keywords:** Cardiopatias Congênitas, Edema Pulmonar, Radiografia Torácica, Insuficiência Cardíaca

## Abstract

**Fundamento:**

O edema pulmonar é uma complicação clinicamente significativa em crianças com cardiopatia congênita (CC). Ocorre com maior frequência em casos complexos e está associado a internações prolongadas. A identificação precoce é essencial devido ao seu impacto direto nos desfechos clínicos.

**Objetivos:**

Avaliar a ultrassonografia pulmonar (USP) como ferramenta diagnóstica para edema pulmonar e identificar fatores clínicos e laboratoriais associados em crianças com CC.

**Métodos:**

Foram incluídas crianças com CC submetidas à USP entre setembro de 2020 e setembro de 2023. Os achados da USP foram comparados com dados clínicos, resultados de radiografia de tórax e níveis de peptídeo natriurético tipo B (BNP). Os pacientes foram classificados de acordo com a categoria fisiopatológica (hiperfluxo pulmonar, hipertensão pulmonar ou outras).

**Resultados:**

Foram avaliadas 62 crianças, com mediana de idade de 5,5 meses (intervalo interquartil, 4-10,1 meses). Hiperfluxo pulmonar foi identificado em 49 pacientes (79%), hipertensão pulmonar em sete (11,3%) e outras condições fisiopatológicas em seis (9,7%). O nível mediano de BNP foi 815,5 pg/mL. O edema pulmonar foi detectado em 36 pacientes (58%) pela radiografia de tórax e em 33 pacientes (53%) pela USP. Observou-se sobreposição significativa, uma vez que 79% dos pacientes com USP positiva também apresentaram edema na radiografia de tórax (p < 0,001). Em contraste, apenas 34,5% dos pacientes com USP negativa apresentaram evidência radiográfica de edema. O edema pulmonar esteve significativamente associado a infecções recorrentes do trato respiratório inferior, baixo peso, índice de massa corporal abaixo de –2 desvios-padrão e níveis elevados de BNP. A análise de regressão logística multivariada identificou falha no crescimento como preditor independente de edema pulmonar. Os achados da radiografia de tórax permaneceram como o principal fator associado ao edema pulmonar.

**Conclusão:**

A USP é um método confiável e livre de radiação para a detecção de edema pulmonar em crianças com CC. A identificação de fatores de risco associados pode facilitar o reconhecimento precoce de pacientes vulneráveis e apoiar intervenções clínicas oportunas e adequadas.

## Graphical abstract

**Figura Central:**
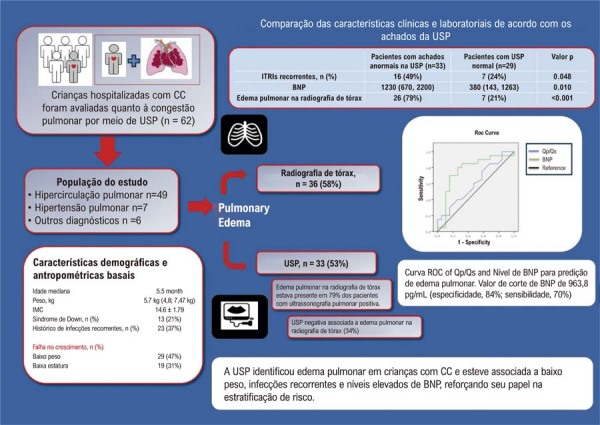
Aprimorando a Avaliação do Edema Pulmonar na Cardiopatia Congênita Pediátrica: O Papel da Ultrassonografia Pulmonar

BNP: peptídeo natriurético tipo B; CC: cardiopatia congênita; IMC: índice de massa corporal; ITRIs: infecções do trato respiratório inferior; ROC: característica de operação do receptor (curva em inglês); USP: ultrassonografia pulmonar.

## Introdução

A identificação precoce e precisa do edema pulmonar é essencial para otimizar os desfechos clínicos em crianças com cardiopatia congênita (CC). A congestão pulmonar contribui para complicações respiratórias, internações prolongadas e deterioração clínica antes da intervenção cirúrgica. Na CC, o edema pulmonar decorre do aumento do fluxo sanguíneo pulmonar, do aumento da pressão hidrostática capilar ou da drenagem linfática prejudicada. Esses mecanismos comprometem a membrana alvéolo-capilar em razão de alterações nas pressões hidrostática e oncótica.^1^

Embora a radiografia de tórax permaneça o método de imagem convencional para avaliar a congestão pulmonar, apresenta sensibilidade limitada e especificidade apenas moderada, o que restringe sua capacidade de detectar sobrecarga hídrica precoce.^2^ Além disso, exames radiográficos repetidos resultam em exposição cumulativa à radiação, o que levanta preocupações quanto à segurança a longo prazo, especialmente em populações pediátricas vulneráveis. Essas limitações têm estimulado a investigação de estratégias alternativas de imagem que permitam a detecção mais precoce e segura do edema pulmonar.

A ultrassonografia pulmonar (USP) tem se destacado como um método de imagem promissor, não invasivo, de baixo custo, reprodutível e livre de radiação, com alta sensibilidade para detectar congestão pulmonar.^3 , 4^ Sua aplicabilidade à beira leito e a capacidade de avaliação dinâmica e em tempo real da aeração pulmonar a tornam particularmente adequada para a prática pediátrica. Além do papel diagnóstico, a USP pode auxiliar na determinação da necessidade e da urgência de intervenções terapêuticas e fornecer informações prognósticas. Embora a USP já tenha sido explorada no manejo em terapia intensiva de crianças com CC, ainda são necessárias evidências adicionais para consolidar seu desempenho diagnóstico na avaliação da congestão pulmonar.^5 - 7^

O objetivo principal deste estudo foi avaliar o edema pulmonar por meio da USP em crianças com CC e correlacionar seus achados com a radiografia de tórax e parâmetros clínicos relevantes, incluindo peptídeo natriurético tipo B (BNP), índice de massa corporal (IMC) e histórico de recorrência de infecções do trato respiratório inferior (ITRIs). Ao examinar essas associações, buscou-se esclarecer se a USP pode atuar como método de imagem complementar ou alternativo na avaliação pré-operatória da congestão pulmonar nessa população pediátrica de alto risco.

## Métodos

### Delineamento do estudo

Este estudo transversal retrospectivo foi conduzido no Hospital X entre setembro de 2020 e setembro de 2023. O objetivo foi investigar a associação entre os achados da USP e a apresentação clínica, a radiografia de tórax e os parâmetros laboratoriais em crianças com diagnóstico de CC. A aprovação ética foi obtida junto ao Comitê de Ética em Pesquisa com Seres Humanos local (Aprovação nº 2023/19-01).

### População do estudo e coleta de dados

Foram incluídos pacientes pediátricos hospitalizados com CC que realizaram USP como parte da avaliação clínica por suspeita de congestão pulmonar. Os pacientes estavam internados na unidade de internação ou foram avaliados no pronto-socorro devido à deterioração clínica. Todos os pacientes elegíveis que atenderam aos critérios de inclusão durante o período do estudo foram incluídos na análise. Devido ao delineamento observacional retrospectivo, não foi realizado cálculo prévio do tamanho da amostra nem análise de poder estatístico.

Dados demográficos predefinidos, características clínicas e achados diagnósticos foram obtidos a partir dos prontuários hospitalares. Os critérios de exclusão incluíram histórico de prematuridade, sepse, hipertensão pulmonar idiopática ou primária, síndrome do desconforto respiratório agudo, pneumonia, doença pulmonar intersticial e outras condições que afetassem a hemodinâmica (por exemplo, insuficiência hepática ou renal).

Foram registradas as seguintes variáveis: sexo, estatura, peso, IMC, presença de síndromes genéticas, uso de medicações e intervenções cirúrgicas prévias. A CC foi categorizada em três grupos fisiopatológicos: hiperfluxo pulmonar, hipertensão pulmonar e outras condições ( Tabela 1 ). Anemia e hipoalbuminemia foram definidas de acordo com valores de referência específicos para a idade. Hiponatremia foi definida como sódio sérico < 135 mmol/L.

**Tabela 1:** – Classificação da cardiopatia congênita de acordo com o mecanismo fisiopatológico

Categoria fisiopatológica	Diagnóstico	n
Hiperfluxo pulmonar (n = 49)	Comunicação interventricular	29
	Defeito do septo atrioventricular	11
	Persistência do canal arterial	4
	Drenagem anômala total das veias pulmonares	4
	Transposição corrigida congenitamente das grandes artérias com insuficiência grave da valva atrioventricular	1
Hipertensão pulmonar (n = 7)	Hipertensão pulmonar secundária	5
	Hipertensão venosa pulmonar (obstrução venosa pulmonar pós-operatória; coarctação da aorta pós-operatória com estenose mitral)	2
Outros (n = 6)	Atresia tricúspide	3
	Tetralogia de Fallot	2
	Atresia pulmonar	1

### Radiografia de tórax e ultrassonografia pulmonar

Todos os pacientes, exceto dois casos pós-operatórios com hipertensão venosa pulmonar, foram avaliados no período pré-operatório. O momento da realização dos exames de imagem (USP e radiografia de tórax) não foi padronizado.

Os exames ultrassonográficos e a interpretação das radiografias de tórax foram realizados por um radiologista pediátrico experiente, que estava cego para os diagnósticos, as características clínicas e os demais resultados de imagem dos pacientes.

As radiografias de tórax foram avaliadas quanto a sinais de edema pulmonar e congestão vascular, incluindo cefalização dos vasos pulmonares, cardiomegalia, linhas B de Kerley, espessamento perivascular, derrame pleural, opacidade hilar, proeminência do cone pulmonar e espessamento da parede brônquica.^8^

Os exames de USP foram realizados em modo B, utilizando transdutor linear (7,7-10 MHz) em equipamento LOGIQ™ P9 (GE HealthCare, Chicago, IL, EUA). Cada hemitórax foi dividido em três regiões anatômicas (anterior, lateral e posterior), utilizando as linhas paraesternal, axilar anterior e axilar posterior como referências.

O edema pulmonar foi considerado presente quando foi identificado mais de um sítio positivo por hemitórax, definido como mais de três linhas B bem espaçadas ou presença de linhas B coalescentes.

### Ecocardiografia

A avaliação ecocardiográfica incluiu a mensuração do diâmetro diastólico final do ventrículo esquerdo (VE) e a estimativa da pressão sistólica do ventrículo direito (PSVD).^9^ Em 39 crianças com shunts esquerda-direita, foi calculada a razão entre fluxo pulmonar e fluxo sistêmico (Qp/Qs).^10^

Todos os pacientes com hipertensão pulmonar apresentavam defeitos cardíacos congênitos subjacentes. A hipertensão pulmonar foi definida como velocidade máxima do jato de regurgitação tricúspide > 3,0 m/s ao Doppler ecocardiográfico.^11^

A hipercirculação pulmonar foi definida por evidência ecocardiográfica de dilatação do VE associada a aumento do fluxo arterial pulmonar, especificamente velocidade de pico > 1,8 m/s na artéria pulmonar principal.^12^

### Diagnóstico de edema pulmonar

O diagnóstico de edema pulmonar foi estabelecido com base em avaliação clínica abrangente. Deterioração clínica, achados respiratórios, hipóxia e sinais de sobrecarga hídrica foram considerados em conjunto com os achados da USP e da radiografia de tórax para definição diagnóstica.

### Análise estatística

A análise estatística foi realizada utilizando o *software* SPSS Statistics for Windows, versão 15 (SPSS Inc., Chicago, IL, EUA).

As variáveis contínuas foram avaliadas quanto à normalidade por meio do teste de Shapiro-Wilk, histogramas e gráficos Q-Q. Variáveis com distribuição normal foram apresentadas como média ± desvio-padrão (DP), e aquelas com distribuição não normal como mediana e intervalo interquartil. As variáveis categóricas foram expressas em frequências e porcentagens e comparadas por meio do teste do qui-quadrado de Pearson ou do teste exato de Fisher, conforme apropriado.

Foram realizadas regressão logística binária e análise da curva característica de operação do receptor (ROC, na sigla em inglês) para avaliar associações entre achados de imagem e parâmetros clínicos e identificar preditores independentes de edema pulmonar. Valores de p < 0,05 foram considerados estatisticamente significativos.

## Resultados

### Características demográficas e clínicas

Um total de 62 crianças foi incluído na análise, com mediana de idade de 5,5 meses (intervalo interquartil, 4-10,1 meses). As características clínicas estão resumidas na Tabela 2 .

**Tabela 2:** – Características demográficas, antropométricas, laboratoriais e de imagem basais da população do estudo

Característica	Valor
**Sexo, n (%)**	
Feminino	25 (40%)
Masculino	37 (60%)
**Estatura, cm, mediana (IQR)**	62 (59,0-68,0)
**Peso, kg, mediana (IQR)**	5,7 (4,8-7,47)
**IMC, média ± DP**	14,6 ± 1,79
**Histórico de infecção recorrente, n (%)**	23 (37%)
**Falha no crescimento, n (%)**	
Baixo peso*	29 (47%)
Baixa estatura^†^	19 (31%)
**Achados laboratoriais**	
Albumina, g/dl, média ± DP	4,2 ± 0,38
Hemoglobina, g/dl, média ± DP	12,7 ± 2
BNP, pg/ml, mediana (IQR)	815,5 (301-1724)
TSH, mIU/ml, mediana (IQR)^‡^	4,02 (3,1-5,2)
T_4_ livre, pmol/l, média ± DP^‡^	19,9 ± 2,7
**Achados na radiografia de tórax**	
Número de radiografias de tórax antes da operação, mediana (IQR)	5 (2,0-10,0)
Edema pulmonar na radiografia de tórax, n (%)	36 (58%)
**Achados ecocardiográficos**	
PSVD, mmHg, média ± DP	51,3 ± 20,8
Qp/Qs, mediana (IQR)	2,1 (1,67-2,3)

*Baixo peso definido como peso para idade < −2 DPs. ^†^Baixa estatura definida como estatura para idade < −2 DPs. ^‡^Valores hormonais tireoidianos disponíveis apenas para crianças com hipotireoidismo. BNP: peptídeo natriurético tipo B; DP: desvio-padrão; IQR: intervalo interquartil; IMC: índice de massa corporal; PSVD: pressão sistólica do ventrículo direito; Qp/Qs: razão entre fluxo pulmonar e fluxo sistêmico; T_4_: tiroxina; TSH: hormônio estimulador da tireoide.

Um total de 13 crianças apresentavam trissomia 21, e um paciente apresentava características dismórficas sem diagnóstico genético confirmado. Oito pacientes tinham hipotireoidismo, todos com síndrome de Down e em uso de levotiroxina.

A fisiopatologia da CC foi classificada como hiperfluxo pulmonar (n = 48), hipertensão pulmonar (n = 7) e outras condições (n = 6) ( Tabela 1 ). Cinco pacientes do grupo com hipertensão pulmonar estavam em uso de terapia específica (sildenafila e/ou antagonistas dos receptores de endotelina). No total, 48 pacientes (77%) estavam em uso de terapia descongestiva (captopril e/ou furosemida), enquanto 11 não utilizavam medicação.

Quatro pacientes haviam sido submetidos previamente a intervenção cirúrgica: dois realizaram correção de drenagem anômala total das veias pulmonares, um foi submetido a procedimento de shunt por atresia pulmonar e um a procedimento de shunt por atresia tricúspide.

Os achados laboratoriais estão apresentados na Tabela 2 . Hipoalbuminemia foi observada em 19 pacientes (31%), hiponatremia em 13 (21%) e anemia em sete (11%).

### Achados radiológicos e ultrassonográficos

**Figura 1:**
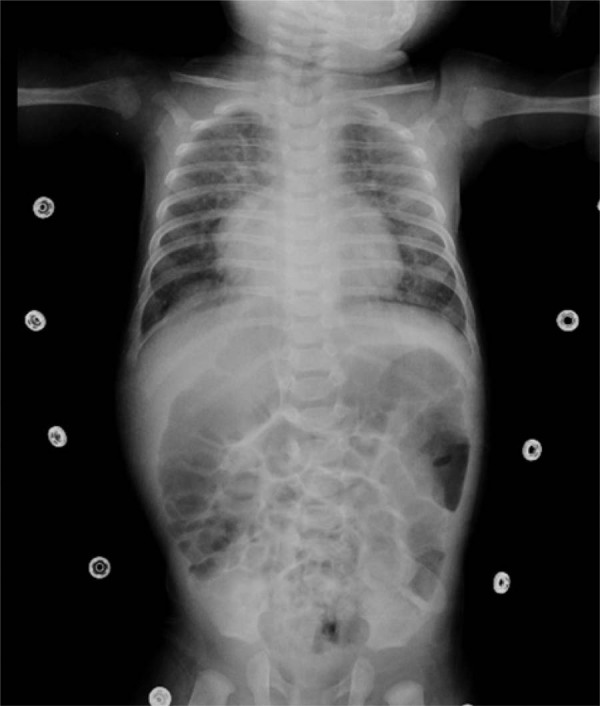
– Radiografia de tórax em incidência póstero-anterior de paciente com edema pulmonar secundário à insuficiência cardíaca. Os achados de imagem incluem cardiomegalia, espessamento peribrônquico/perivascular, aumento difuso bilateral das opacidades pulmonares, opacificação hilar bilateral e sinais compatíveis com edema alveolar.

Os achados radiográficos estão resumidos nas Tabelas 2 e 3. O edema pulmonar foi frequentemente observado ( Figura 1 ), comumente acompanhado de cardiomegalia, linhas B de Kerley e opacificação hilar bilateral.

A ecocardiografia demonstrou aumento da pressão arterial pulmonar em 43 pacientes (69%). Entre as crianças com *shunts* esquerda-direita, dilatação do VE foi observada em 27 (43,5%).

**Figura 2:**
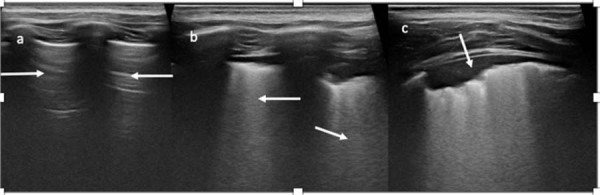
– Achados representativos da ultrassonografia pulmonar no edema pulmonar. a) Linhas A dominantes na zona superior do pulmão esquerdo (setas). (b) Linhas B confluentes na zona média do pulmão esquerdo (setas). (c) Pequena consolidação periférica na zona inferior do pulmão direito (seta) associada a linhas B que se misturam.

A USP demonstrou achados anormais em 33 pacientes (53%) ( Figura 2 ). Entre esses, 13 (39%) eram do sexo feminino e 20 (61%) do sexo masculino. Síndrome de Down esteve presente em 21% dos pacientes com USP alterada, e hipotireoidismo em 12%. Histórico de ITRIs foi significativamente mais comum entre crianças com achados anormais na USP. Um resumo comparativo dos parâmetros clínicos, laboratoriais e ecocardiográficos entre pacientes com e sem alterações na USP está apresentado na Tabela 3 .

**Tabela 3:** – Comparação das características clínicas e laboratoriais em crianças com e sem achados anormais na USP

Variável	USP anormal (n = 33)	USP normal (n = 29)	Valor p
Idade, meses, mediana (IQR)	6 (4-9)	5 (4-12)	0,576
Peso, kg, mediana (IQR)	5,4 (4,65-7,0)	6,2 (5,0-8,3)	0,646
Falha no crescimento, n (%)	22 (68%)	11 (38%)	**0,024**
Baixo peso, n (%)	19 (58%)	8 (29%)	**0,023**
Baixa estatura, n (%)	10 (31%)	7 (24%)	0,536
IMC, média ± DP	14,3 ± 2,0	15,1 ± 1,4	0,099
Hipotireoidismo, n (%)	4 (12%)	4 (14%)	1,000
ITRI recorrente, n (%)	16 (49%)	7 (24%)	**0,048**
Hiponatremia, n (%)	7 (21%)	6 (21%)	0,960
Anemia, n (%)	6 (18%)	2 (7%)	0,264
Hipoalbuminemia, n (%)	11 (33%)	6 (21%)	0,265
BNP, pg/mL, mediana (IQR)	1.230 (670-2.200)	380 (143-1.263)	**0,010**
Edema pulmonar na radiografia de tórax, n (%)	26 (79%)	7 (21%)	**< 0,001**
PSVD elevada, n (%)	22 (67%)	21 (72%)	0,624
PSVD, mmHg, média ± DP	53 ± 20	45 ± 13	0,488
Dilatação do VE, n (%)	14 (42%)	13 (45%)	0,849
Qp/Qs, mediana (IQR)	2,1 (1,7-2,4)	1,8 (1,6-2,3)	0,260

Valores de p estatisticamente significativos estão destacados em negrito. Variáveis contínuas foram analisadas pelo teste de Mann-Whitney. Variáveis categóricas foram comparadas pelo teste do qui-quadrado de Pearson ou pelo teste exato de Fisher, conforme apropriado. DP: desvio-padrão; IMC: índice de massa corporal; IQR: intervalo interquartil; ITRI: infecção do trato respiratório inferior; BNP: peptídeo natriurético tipo B; PSVD: pressão sistólica do ventrículo direito; VE: ventrículo esquerdo; Qp/Qs: razão entre fluxo pulmonar e fluxo sistêmico.

Entre as crianças com edema pulmonar identificado pela USP, 79% também apresentavam evidência radiográfica de edema pulmonar na radiografia de tórax. Em contraste, 34,5% dos pacientes com USP normal apresentaram edema pulmonar na radiografia. Essa diferença foi estatisticamente significativa (p < 0,001).

Não foi observada diferença estatisticamente significativa na frequência de achados anormais na USP entre pacientes em uso de terapia descongestiva e aqueles sem tratamento. Além disso, não foi encontrada associação significativa entre alterações na USP e parâmetros ecocardiográficos, incluindo Qp/Qs, dilatação do VE e PSVD.

A proporção de crianças com baixo peso foi significativamente maior no grupo com USP alterada. ITRIs recorrentes também foram mais frequentes entre pacientes com edema pulmonar identificado pela USP.

**Figura 3:**
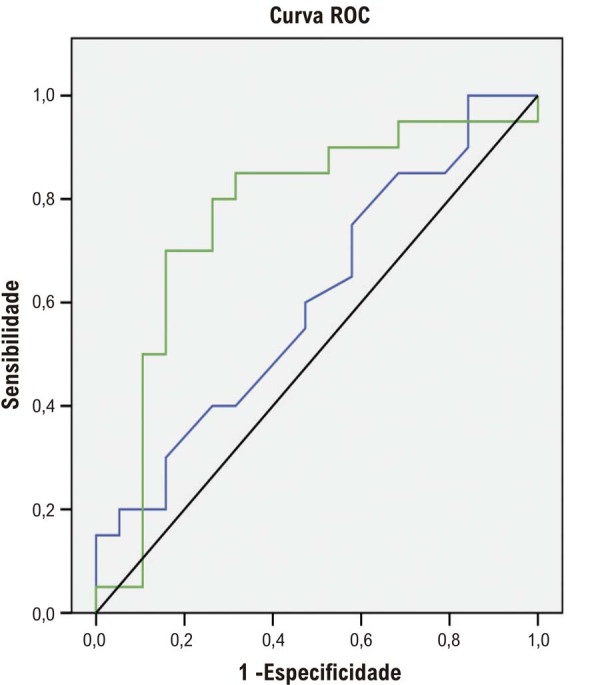
– Curvas ROC para a razão Qp/Qs e os níveis de BNP na predição de edema pulmonar. A curva verde representa o BNP, e a curva azul representa a razão Qp/Qs. A linha preta diagonal indica a linha de não discriminação. A área sob a curva (AUC) foi maior para o BNP. Um valor de corte de BNP de 963,8 pg/mL apresentou sensibilidade de 70% e especificidade de 84%.

A análise da curva ROC demonstrou que o BNP foi preditor significativo de edema pulmonar na USP. Um valor de corte de 963,8 pg/mL apresentou sensibilidade de 70% e especificidade de 84% ( Figura 3 ; Figura Central ). A área sob a curva foi 0,768 (intervalo de confiança de 95%, 0,607-0,930; p = 0,004).

### Análise multivariada

Foi realizada regressão logística multivariada para identificar preditores independentes de edema pulmonar. O modelo global foi estatisticamente significativo segundo o teste Omnibus dos coeficientes do modelo (χ^2^ = 23,784; graus de liberdade = 5; p < 0,001). O valor de R^2^ de Nagelkerke foi 0,425, indicando que o modelo explicou 42,5% da variância. O teste de ajuste de Hosmer-Lemeshow não foi estatisticamente significativo (p = 0,177), indicando bom ajuste do modelo.

As variáveis incluídas no modelo foram histórico de ITRIs recorrentes, falha no crescimento, uso de terapia descongestiva, hipoalbuminemia e presença de edema pulmonar na radiografia de tórax ( Tabela 4 ).

**Tabela 4:** – Análise de regressão logística das variáveis associadas ao edema pulmonar

Variável	OR (IC 95%)	Valor p
ITRI recorrente	0,325 (0,084-1,26)	0,104
Falha no crescimento	0,217 (0,058-0,818)	**0,024**
Terapia descongestiva	4,605 (0,817-25,96)	0,084
Hipoalbuminemia	2,043 (0,376-11,11)	0,408
Edema pulmonar na radiografia de tórax	10,05 (2,6-38,83)	**< 0,01**

Valores de p estatisticamente significativos estão destacados em negrito. IC: intervalo de confiança; ITRI: infecção do trato respiratório inferior; OR: odds ratio.

Falha no crescimento esteve significativamente e negativamente associada à ocorrência de edema pulmonar. Em contraste, a presença de edema pulmonar na radiografia de tórax esteve fortemente associada ao aumento do risco. ITRIs recorrentes, uso de terapia descongestiva e hipoalbuminemia não foram preditores estatisticamente significativos no modelo multivariado.

## Discussão

Este estudo destaca o potencial diagnóstico da USP na detecção de edema pulmonar em crianças com CC. Achados anormais na USP estiveram significativamente associados à evidência radiográfica de congestão pulmonar, bem como a importantes indicadores clínicos, incluindo baixo peso, ITRIs e níveis elevados de BNP ( Figura Central ). A síndrome de Down não esteve significativamente associada a alterações na USP nesta coorte. A análise multivariada demonstrou que a presença de edema pulmonar na radiografia de tórax e falha no crescimento foram preditores independentes de edema pulmonar. Esses achados reforçam o papel da USP como método de imagem prático e livre de radiação para identificação precoce de complicações pulmonares nessa população vulnerável.

A USP é uma ferramenta valiosa para avaliar a aeração e a complacência pulmonar em crianças com CC, que podem estar comprometidas devido à lesão por reperfusão ou à redução da perfusão pulmonar. O método permite monitoramento em tempo real da aeração pulmonar e avaliação da resposta ao tratamento.^13^ A presença de linhas B na USP reflete aumento do conteúdo de água extravascular pulmonar e aumento da relação ar-líquido no interstício pulmonar.^4^

Estudos prévios, incluindo o de Wu et al.,^14^ demonstraram sensibilidade e especificidade superiores da USP em comparação com a radiografia de tórax na detecção de edema pulmonar. No entanto, nossos achados sugerem uma relação mais complexa entre esses métodos de imagem. Aproximadamente 34,5% dos pacientes com USP normal apresentaram congestão pulmonar na radiografia de tórax. Diferentemente do estudo de Wu et al.,^14^ no qual os pacientes foram avaliados no período pré-operatório, uma proporção substancial de nossa coorte estava em uso de terapia descongestiva. Melhorias hemodinâmicas e alveolares relacionadas ao tratamento podem ter influenciado os achados da USP, enquanto alterações radiográficas podem persistir apesar da melhora clínica.^4^ Essas observações sugerem que, embora a USP seja um método confiável para diferenciar edema pulmonar de origem cardíaca e não cardíaca, o uso combinado de radiografia de tórax e USP pode aumentar a acurácia diagnóstica em crianças com CC.

Crianças com CC são vulneráveis a lesão pulmonar independentemente da presença de hiperfluxo pulmonar. Os mecanismos incluem disfunção do surfactante, edema pulmonar, extravasamento capilar, desequilíbrio ventilação-perfusão e aumento da resistência das vias aéreas.^15 , 16^ Quando ITRIs ocorrem nessa população, estão associadas a pior prognóstico, incluindo hospitalizações mais frequentes, internações prolongadas e maior risco de mortalidade.^15^ Em nosso estudo, ITRIs recorrentes estiveram associadas a maior prevalência de edema pulmonar na USP. Esses achados indicam que crianças com ITRIs recorrentes requerem monitoramento rigoroso para reduzir o risco de complicações pulmonares. Além disso, a adesão rigorosa aos programas de imunização contra patógenos respiratórios pode ser particularmente relevante em lactentes com CC, a fim de mitigar desfechos adversos.

Alterações nos hormônios tireoidianos podem afetar a função cardíaca sistólica e diastólica por meio de mudanças no controle do cálcio, podendo levar à redução do desempenho cardíaco.^17 , 18^ Embora o hipotireoidismo seja reconhecido como fator contribuinte para insuficiência cardíaca, não foi observada associação entre hipotireoidismo e achados anormais na USP nesta coorte. Isso pode refletir reposição hormonal adequada e manutenção do estado eutireoidiano nos pacientes afetados. Pesquisas anteriores demonstraram associação entre hormônio estimulador da tireoide e níveis de BNP, o que sugere possíveis efeitos sobre a função cardíaca.^18^ Investigações adicionais sobre o estado tireoidiano em crianças com CC que apresentam deterioração clínica podem ser justificadas.

Níveis elevados de BNP estiveram significativamente associados a achados anormais na USP. O BNP é um biomarcador consolidado que reflete gravidade da insuficiência cardíaca, magnitude do shunt e sobrecarga volumétrica ventricular na CC.^19^ Kaya et al.^6^ também relataram associação significativa entre alterações na USP, aumento do proBNP, necessidade de diuréticos e suporte respiratório. Em populações em diálise, foi proposto um ponto de corte de BNP de 165 pg/mL para identificar congestão pulmonar confirmada pela USP.^20^ Em nossa coorte, um valor de BNP > 963,8 pg/mL previu edema pulmonar na USP com sensibilidade de 70% e especificidade de 84%.

A desnutrição em crianças com CC está associada a desfechos adversos, incluindo maior mortalidade, internações prolongadas e maior tempo de permanência em unidade de terapia intensiva no período pré-operatório.^21 , 22^ Aproximadamente metade dos pacientes deste estudo apresentava baixo peso. O edema pulmonar detectado pela USP foi mais frequente entre crianças com baixo peso e IMC para a idade abaixo de −2 DPs. Embora estudos anteriores não tenham demonstrado impacto significativo do IMC nos desfechos da CC,^22^ nossos achados sugerem que o estado nutricional pode influenciar a presença de edema pulmonar. A avaliação abrangente de parâmetros antropométricos pode, portanto, contribuir para melhor estratificação de risco e previsão prognóstica.

Algumas limitações devem ser consideradas. Trata-se de estudo retrospectivo, unicêntrico, com tamanho amostral relativamente pequeno e heterogeneidade clínica quanto ao tipo de CC, idade, uso de medicações e comorbidades. O momento da realização dos exames de imagem não foi padronizado, o que pode ter influenciado a detecção de congestão pulmonar. Embora a maioria dos pacientes tenha sido avaliada no período pré-operatório, dois casos pós-operatórios foram incluídos. Apesar dessas limitações, o estudo oferece achados preliminares que podem orientar o delineamento de futuras investigações prospectivas. O viés do observador foi minimizado por meio da interpretação cega das imagens.

## Conclusão

Este estudo avaliou o papel da USP na detecção de edema pulmonar em crianças com CC. Achados anormais na USP estiveram significativamente associados a baixo peso, ITRIs recorrentes e níveis elevados de BNP, o que reforça seu valor na identificação de pacientes de alto risco. A síndrome de Down não esteve associada a alterações na USP, o que indica que não predispõe independentemente à congestão pulmonar detectável por meio da USP.

Devido à sua aplicabilidade à beira do leito e à ausência de exposição à radiação, a USP parece ser uma modalidade complementar útil à radiografia de tórax na avaliação pré-operatória da congestão pulmonar em crianças com CC. Estudos prospectivos, multicêntricos e com maior tamanho amostral são necessários para padronizar protocolos de USP, validar seu desempenho diagnóstico e determinar seu impacto na tomada de decisão clínica e nos desfechos dos pacientes em diferentes subtipos de CC.
