# Supplementary material for: Aprimorando a Avaliação do Edema Pulmonar na Cardiopatia Congênita Pediátrica: O Papel da Ultrassonografia Pulmonar
Source: Arq Bras Cardiol. 2026 Apr 14;123(3):e20250322. [Article in Portuguese] doi: 10.36660/abc.20250322 (PMC13128202; doi:10.36660/abc.20250322)
Supplement: Translation

# Translation: Improving Pulmonary Edema Assessment in Pediatric Congenital Heart Disease: The Role of Lung Ultrasonography

**DOI:** 10.36660/abc.20250322i

**Authors:** Yasemin Nuran Donmez, Derya Bako, Safak Alpat, Dilek Giray, Serdar Epcacan

**Affiliations:** 1 Türkiye Van Training and Research Hospital, Van – Türkiye

**Keywords:** Congenital Heart Defects, Pulmonary Edema, Thoracic Radiography, Heart Failure

## Abstract

**Background:**

Pulmonary edema is a clinically significant complication in children with congenital heart disease (CHD). It occurs more frequently in complex cases and is associated with prolonged hospital stays. Early identification is essential because of its direct impact on clinical outcomes.

**Objectives:**

To evaluate lung ultrasonography (LUS) as a diagnostic tool for pulmonary edema and to identify associated clinical and laboratory factors in children with CHD.

**Methods:**

Children with CHD who underwent LUS between September 2020 and September 2023 were included. LUS findings were compared with clinical data, chest radiography results, and B-type natriuretic peptide (BNP) levels. Patients were classified according to pathophysiological category (pulmonary overflow, pulmonary hypertension, or other).

**Results:**

A total of 62 children were evaluated, with a median age of 5.5 months (interquartile range, 4-10.1 months). Pulmonary overflow was identified in 49 patients (79%), pulmonary hypertension in seven (11.3%), and other pathophysiological conditions in six (9.7%). The median BNP level was 815.5 pg/mL. Pulmonary edema was detected in 36 patients (58%) by chest radiography and in 33 patients (53%) by LUS. A significant overlap was observed, as 79% of patients with positive LUS findings also demonstrated edema on chest radiography (p < 0.001). In contrast, only 34.5% of patients with negative LUS findings had radiographic evidence of edema. Pulmonary edema was significantly associated with recurrent lower respiratory tract infections, underweight status, body mass index below –2 standard deviations, and increased BNP levels. Multivariate logistic regression analysis identified failure to thrive as an independent predictor of pulmonary edema. Chest radiography findings remained the primary factor associated with pulmonary edema.

**Conclusion:**

LUS is a reliable, radiation-free modality for detecting pulmonary edema in children with CHD. Identification of associated risk factors may facilitate early recognition of vulnerable patients and support timely, appropriate clinical interventions.

## Introduction

Early, accurate identification of pulmonary edema is essential for optimizing clinical outcomes in children with congenital heart disease (CHD). Pulmonary congestion contributes to respiratory complications, prolonged hospitalization, and clinical deterioration prior to surgical intervention. In CHD, pulmonary edema arises from increased pulmonary blood flow, increased capillary hydrostatic pressure, or impaired lymphatic drainage. These mechanisms disrupt the alveolar-capillary membrane due to alterations in hydrostatic and oncotic pressures.^1^

Although chest radiography remains the conventional imaging modality for assessing pulmonary congestion, it has limited sensitivity and only moderate specificity, which restrict its ability to detect early fluid overload.^2^Furthermore, repeated radiographic examinations result in cumulative radiation exposure, raising concerns about long-term safety, particularly in vulnerable pediatric populations. These limitations have prompted investigation into alternative imaging strategies that allow earlier, safer detection of pulmonary edema.

Lung ultrasonography (LUS) has emerged as a promising noninvasive, low-cost, repeatable, and radiation-free imaging modality with high sensitivity for detecting pulmonary congestion.^3 , 4^Its bedside applicability and capacity for dynamic, real-time assessment of lung aeration make it particularly well suited for pediatric practice. Beyond its diagnostic role, LUS may assist in determining the need and urgency of therapeutic interventions and may provide prognostic information. Although LUS has been explored in the critical care management of children with CHD, additional evidence is required to further establish its diagnostic performance in the assessment of pulmonary congestion.^5 - 7^

The primary objective of this study was to evaluate pulmonary edema using LUS in children with CHD and to correlate LUS findings with chest radiography and relevant clinical parameters, including B-type natriuretic peptide (BNP), body mass index (BMI), and a history of recurrent lower respiratory tract infections (LRTIs). By examining these associations, we sought to clarify whether LUS may serve as a complementary or alternative imaging modality for the preoperative assessment of pulmonary congestion in this high-risk pediatric population.

## Methods

### Study design

This retrospective cross-sectional study was conducted at X Hospital between September 2020 and September 2023. The objective was to investigate the association between LUS findings and clinical presentation, chest radiography, and laboratory parameters in children diagnosed with CHD. Ethical approval was obtained from the local Human Research Ethics Committee (Approval No. 2023/19-01).

### Study population and data collection

Hospitalized pediatric patients with CHD who underwent LUS as part of the clinical evaluation for suspected pulmonary congestion were included. Patients were either admitted to the inpatient unit or evaluated in the emergency department due to clinical deterioration. All eligible patients who met the inclusion criteria during the study period were included in the analysis. Because of the retrospective observational design, no prior sample size calculation or power analysis was performed.

Predefined demographic data, clinical characteristics, and diagnostic findings were retrieved from hospital medical records. Exclusion criteria included a history of prematurity, sepsis, idiopathic or primary pulmonary hypertension, acute respiratory distress syndrome, pneumonia, interstitial lung disease, and other conditions affecting hemodynamics (e.g., liver or kidney failure).

The following variables were recorded: sex, height, weight, BMI, presence of genetic syndromes, medications, and previous surgical interventions. CHD was categorized into three pathophysiological groups: pulmonary overflow, pulmonary hypertension, and other conditions ( Table 1 ). Anemia and hypoalbuminemia were defined according to age-specific reference ranges. Hyponatremia was defined as a serum sodium level < 135 mmol/L.

**Table 1:** – Classification of congenital heart disease according to pathophysiological mechanism

Pathophysiological category	Diagnosis	n
Pulmonary overflow (n = 49)	Ventricular septal defect	29
	Atrioventricular septal defect	11
	Patent ductus arteriosus	4
	Total anomalous pulmonary venous return	4
	Congenitally corrected transposition of the great arteries with severe atrioventricular valve regurgitation	1
Pulmonary hypertension (n = 7)	Secondary pulmonary hypertension	5
	Pulmonary venous hypertension (postoperative pulmonary venous obstruction; postoperative coarctation of the aorta with mitral stenosis)	2
Other (n = 6)	Tricuspid atresia	3
	Tetralogy of Fallot	2
	Pulmonary atresia	1

### Chest radiography and lung ultrasonography

All patients, except two postoperative cases with pulmonary venous hypertension, were evaluated in the preoperative period. The timing of imaging (LUS and chest radiography) was not standardized.

Sonographic examinations and chest radiograph interpretations were performed by an experienced pediatric radiologist who was blinded to patients’ diagnoses, clinical characteristics, and other imaging results.

Chest radiographs were assessed for signs of pulmonary edema and vascular congestion, including cephalization of pulmonary vessels, cardiomegaly, Kerley B lines, perivascular cuffing, pleural effusion, hilar haziness, prominence of the pulmonary conus, and bronchial wall thickening.^8^

LUS examinations were performed using B-mode imaging with a linear transducer (7.7-10 MHz) on a LOGIQ™ P9 ultrasound system (GE HealthCare, Chicago, IL, USA). Each hemithorax was divided into three anatomical regions (anterior, lateral, and posterior), using the parasternal, anterior axillary, and posterior axillary lines as landmarks.

Pulmonary edema was considered present when more than one positive site per hemithorax was identified, defined as more than three well-spaced B-lines or the presence of coalescent B-lines.

### Echocardiography

Echocardiographic evaluation included measurement of left ventricular (LV) end-diastolic diameter and estimation of right ventricular systolic pressure (RVSP).^9^In 39 children with left-to-right shunts, the pulmonary-to-systemic blood flow ratio (Qp/Qs) was calculated.^10^

All patients with pulmonary hypertension had underlying congenital heart defects. Pulmonary hypertension was defined as a peak tricuspid regurgitation velocity > 3.0 m/s on Doppler echocardiography.^11^

Pulmonary overcirculation was defined by echocardiographic evidence of LV dilatation accompanied by increased pulmonary arterial flow, specifically a peak velocity > 1.8 m/s in the main pulmonary artery.^12^

### Diagnosis of pulmonary edema

Pulmonary edema was diagnosed based on comprehensive clinical evaluation. Clinical deterioration, respiratory findings, hypoxia, and signs of fluid overload were considered in conjunction with LUS and chest radiography findings to establish the diagnosis.

### Statistical analysis

Statistical analysis was performed using the SPSS Statistics for Windows, version 15 (SPSS Inc., Chicago, Ill., USA).

Continuous variables were assessed for normality using the Shapiro-Wilk test, histograms, and Q-Q plots. Normally distributed variables were presented as mean ± standard deviation (SD), and non-normally distributed variables as median with interquartile range. Categorical variables were expressed as frequencies and percentages and compared using Pearson’s chi-square test or Fisher’s exact test, as appropriate.

Binary logistic regression and receiver operating characteristic (ROC) curve analysis were performed to evaluate associations between imaging findings and clinical parameters and to identify independent predictors of pulmonary edema. A p-value < 0.05 was considered statistically significant.

## Results

### Demographic and clinical features

A total of 62 children were included in the analysis, with a median age of 5.5 months (interquartile range, 4-10.1 months). Clinical characteristics are summarized in Table 2 .

**Table 2:** – Baseline demographic, anthropometric, laboratory, and imaging characteristics of the study population

Characteristic	Value
**Sex, n (%)**	
Women	25 (40%)
Men	37 (60%)
**Height, cm, median (IQR)**	62 (59.0-68.0)
**Weight, kg, median (IQR)**	5.7 (4.8-7.47)
**BMI, mean ± SD**	14.6 ± 1.79
**Recurrent infection history, n (%)**	23 (37%)
**Failure to thrive, n (%)**	
Underweight*	29 (47%)
Stunting^†^	19 (31%)
**Laboratory findings**	
Albumin, g/dL, mean ± SD	4.2 ± 0.38
Hemoglobin, g/dL, mean ± SD	12.7 ± 2
BNP, pg/mL, median (IQR)	815.5 (301-1724)
TSH, mIU/mL, median (IQR)^‡^	4.02 (3.1-5.2)
Free T4, pmol/L, mean ± SD^‡^	19.9 ± 2.7
**Chest radiography findings**	
Number of chest radiographies before operation, median (IQR)	5 (2.0-10.0)
Pulmonary edema on chest radiography, n (%)	36 (58%)
**Echocardiographic findings**	
RVSP, mmHg, mean ± SD	51.3 ± 20.8
Qp/Qs, median (IQR)	2.1 (1.67-2.3)

*Underweight defined as weight-for-age < −2 SDs.^†^Stunting defined as height-for-age < −2 SDs.^‡^Thyroid hormone values were available only for children with hypothyroidism. BMI: body mass index; BNP: B-type natriuretic peptide; IQR: interquartile range; Qp/Qs: pulmonary-to-systemic blood flow ratio; RVSP: right ventricular systolic pressure; SD: standard deviation; T_4_: thyroxine; TSH: thyroid-stimulating hormone.

A total of 13 children had trisomy 21, and one patient presented with dysmorphic features without a confirmed genetic diagnosis. A total of eight patients had hypothyroidism, all of whom had Down syndrome and were receiving levothyroxine therapy.

CHD pathophysiology was classified as pulmonary overflow (n = 48), pulmonary hypertension (n = 7), and other conditions (n = 6) ( Table 1 ). Five patients in the pulmonary hypertension group were receiving targeted therapy (sildenafil and/or endothelin receptor antagonists). Overall, 48 patients (77%) were receiving anti-congestive therapy (captopril and/or furosemide), whereas 11 were not on any medication.

Four patients had undergone prior surgical intervention: two had repair of total anomalous pulmonary venous return, one had a shunt procedure for pulmonary atresia, and one had a shunt procedure for tricuspid atresia.

Laboratory findings are presented in Table 2 . Hypoalbuminemia was observed in 19 patients (31%), hyponatremia in 13 (21%), and anemia in seven (11%).

### Radiological and ultrasonographic findings

Radiographic findings are summarized in Tables 2 and 3. Pulmonary edema was frequently observed ( Figure 1 ), commonly accompanied by cardiomegaly, Kerley B lines, and bilateral hilar haziness.

**Figure 1:** – Posterior-anterior chest radiograph of a patient with pulmonary edema secondary to heart failure. Imaging findings include cardiomegaly, peribronchovascular cuffing, diffuse bilateral increase in pulmonary opacities, bilateral hilar haziness, and features consistent with alveolar edema.

Echocardiography demonstrated increased pulmonary arterial pressure in 43 patients (69%). Among children with left-to-right shunts, LV dilatation was observed in 27 (43.5%).

LUS demonstrated abnormal findings in 33 patients (53%) ( Figure 2 ). Among these, 13 (39%) were women and 20 (61%) were men. Down syndrome was present in 21% of patients with abnormal LUS findings, and hypothyroidism in 12%. A history of recurrent LRTIs was significantly more common among children with abnormal LUS findings. A comparative summary of clinical, laboratory, and echocardiographic parameters in patients with and without abnormal LUS findings is provided in Table 3 .

**Figure 2:** – Representative lung ultrasonography findings in pulmonary edema. a) Dominant A-lines in the upper zone of the left lung (arrows). (b) Confluent B-lines in the middle zone of the left lung (arrows). (c) Small peripheral consolidation in the lower zone of the right lung (arrow) associated with confluent B-lines.

**Table 3:** – Comparison of clinical and laboratory characteristics in children with and without abnormal LUS findings

Variable	Abnormal LUS (n = 33)	Normal LUS (n = 29)	p-value
Age, months, median (IQR)	6 (4-9)	5 (4-12)	0.576
Weight, kg, median (IQR)	5.4 (4.65-7.0)	6.2 (5.0-8.3)	0.646
Failure to thrive, n (%)	22 (68%)	11 (38%)	**0.024**
Underweight, n (%)	19 (58%)	8 (29%)	**0.023**
Stunting, n (%)	10 (31%)	7 (24%)	0.536
BMI, mean ± SD	14.3 ± 2.0	15.1 ± 1.4	0.099
Hypothyroidism, n (%)	4 (12%)	4 (14%)	1.000
Recurrent LRTI, n (%)	16 (49%)	7 (24%)	**0.048**
Hyponatremia, n (%)	7 (21%)	6 (21%)	0.960
Anemia, n (%)	6 (18%)	2 (7%)	0.264
Hypoalbuminemia, n (%)	11 (33%)	6 (21%)	0.265
BNP, pg/mL, median (IQR)	1,230 (670-2,200)	380 (143-1,263)	**0.010**
Pulmonary edema on chest radiography, n (%)	26 (79%)	7 (21%)	**< 0.001**
High RVSP, n (%)	22 (67%)	21 (72%)	0.624
RVSP, mmHg, mean ± SD	53 ± 20	45 ± 13	0.488
LV dilatation, n (%)	14 (42%)	13 (45%)	0.849
Qp/Qs, median (IQR)	2.1 (1.7-2.4)	1.8 (1.6-2.3)	0.260

Statistically significant p values are shown in bold. Continuous variables were analyzed using the Mann-Whitney U test. Categorical variables were compared using Pearson’s chi-square test or Fisher’s exact test, as appropriate. BMI: body mass index; BNP: B-type natriuretic peptide; IQR: interquartile range; LRTI: lower respiratory tract infection; LUS: lung ultrasonography; LV: left ventricle; Qp/Qs: pulmonary-to-systemic blood flow ratio; RVSP: right ventricular systolic pressure; SD: standard deviation.

Among children with pulmonary edema detected by LUS, 79% also had radiographic evidence of pulmonary edema on chest radiography. In contrast, 34.5% of patients with normal LUS findings demonstrated pulmonary edema on chest radiography. This difference was statistically significant (p < 0.001).

No statistically significant difference in abnormal LUS findings was observed between patients receiving anti-congestive therapy and those not receiving such treatment. Additionally, no significant association was found between abnormal LUS findings and echocardiographic parameters, including Qp/Qs, LV dilatation, and RVSP.

The proportion of underweight children was significantly higher in the group with abnormal LUS findings. Recurrent LRTIs were also more frequent among patients with pulmonary edema identified by LUS.

ROC curve analysis demonstrated that BNP was a significant predictor of pulmonary edema on LUS. A BNP cut-off value of 963.8 pg/mL yielded a sensitivity of 70% and specificity of 84% ( Figure 3 ; Central Illustration ). The area under the curve was 0.768 (95% CI, 0.607-0.930; p = 0.004).

**Figure 3:** – ROC curves for Qp/Qs ratio and BNP levels in predicting pulmonary edema. The green curve represents BNP, and the blue curve represents the Qp/Qs ratio. The diagonal black line indicates the line of no discrimination. The AUC was higher for BNP. A BNP cutoff value of 963.8 pg/mL yielded a sensitivity of 70% and a specificity of 84%.

### Multivariate analysis

Multivariate logistic regression was performed to identify independent predictors of pulmonary edema. The overall model was statistically significant according to the Omnibus test of model coefficients (χ^2^= 23.784, degrees of freedom = 5, p < 0.001). The Nagelkerke R^2^value was 0.425, which indicates that the model explained 42.5% of the variance. The Hosmer-Lemeshow goodness-of-fit test was not statistically significant (p = 0.177), which indicates adequate model fit.

Variables included in the model were history of recurrent LRTIs, failure to thrive, use of anti-congestive therapy, hypoalbuminemia, and presence of pulmonary edema on chest radiography ( Table 4 ).

**Table 4:** – Logistic regression analysis of variables associated with pulmonary edema

Variable	OR (95% CI)	p-value
Recurrent LRTI	0.325 (0.084-1.26)	0.104
Failure to thrive	0.217 (0.058-0.818)	**0.024**
Anti-congestive therapy	4.605 (0.817-25.96)	0.084
Hypoalbuminemia	2.043 (0.376-11.11)	0.408
Pulmonary edema on chest radiography	10.05 (2.6-38.83)	**< 0.01**

Statistically significant p-values are shown in bold. LRTI: lower respiratory tract infection; OR: odds ratio.

Failure to thrive was significantly and negatively associated with pulmonary edema occurrence. In contrast, the presence of pulmonary edema on chest radiography was strongly associated with an increased risk. Recurrent LRTIs, use of anti-congestive therapy, and hypoalbuminemia were not statistically significant predictors in the multivariate model.

## Discussion

This study underscores the diagnostic potential of LUS for detecting pulmonary edema in children with CHD. Abnormal LUS findings were significantly associated with radiographic evidence of pulmonary congestion as well as important clinical indicators, including underweight status, recurrent LRTIs, and increased BNP levels ( Central Illustration ). Down syndrome was not significantly associated with abnormal LUS findings in this cohort. Multivariate analysis demonstrated that chest radiography evidence of pulmonary edema and failure to thrive independently predicted pulmonary edema. These findings support the role of LUS as a practical, radiation-free imaging modality for early identification of pulmonary complications in this vulnerable population.

LUS is a valuable tool for assessing lung aeration and compliance in children with CHD, which may be impaired due to reperfusion injury or reduced pulmonary perfusion. It allows real-time monitoring of lung aeration and evaluation of response to treatment.^13^The presence of B-lines on LUS reflects an increased extravascular lung water content and an increased air-to-fluid ratio within the pulmonary interstitium.^4^

Previous studies, including that of Wu et al.,^14^demonstrated superior sensitivity and specificity of LUS compared with chest radiography in detecting pulmonary edema. However, our findings suggest a more complex relationship between these imaging modalities. Approximately 34.5% of patients with normal LUS findings exhibited pulmonary congestion on chest radiography. In contrast to Wu et al.,^14^whose patients were evaluated preoperatively, a substantial proportion of our cohort was receiving anti-congestive therapy. Treatment-related hemodynamic and alveolar improvements may have influenced LUS findings, whereas radiographic changes can persist despite clinical improvement.^4^These observations suggest that although LUS is a reliable modality for distinguishing cardiac from non-cardiac pulmonary edema, combined use of chest radiography and LUS may enhance diagnostic accuracy in children with CHD.

Children with CHD are vulnerable to lung injury regardless of the presence of pulmonary overflow. Mechanisms include impaired surfactant function, pulmonary edema, capillary leak, ventilation-perfusion mismatch, and increased airway resistance.^15 , 16^When LRTIs occur in this population, they are associated with worse prognosis, including more frequent hospitalizations, prolonged hospital stays, and increased mortality risk.^15^In our study, recurrent LRTIs were associated with a higher prevalence of pulmonary edema on LUS. These findings suggest that children with recurrent LRTIs require close monitoring to reduce the risk of pulmonary complications. Furthermore, strict adherence to immunization programs targeting respiratory pathogens may be particularly important in infants with CHD to mitigate adverse outcomes.

Thyroid hormone alterations can affect systolic and diastolic cardiac function through changes in calcium handling, potentially leading to reduced cardiac performance.^17 , 18^Although hypothyroidism is recognized as a contributor to heart failure, no association was observed between hypothyroidism and abnormal LUS findings in our cohort. This may reflect adequate hormone replacement therapy and maintained euthyroid status among affected patients. Prior research has demonstrated associations between thyroid-stimulating hormone and BNP levels, which suggests potential effects on cardiac function.^18^Further investigation into thyroid status in children with CHD experiencing clinical deterioration may be warranted.

Increased BNP levels were significantly associated with abnormal LUS findings. BNP is a well-established biomarker reflecting heart failure severity, shunt magnitude, and ventricular volume overload in CHD.^19^Kaya et al.^6^similarly reported significant associations between abnormal LUS findings, increased proBNP levels, diuretic requirement, and need for respiratory support. In dialysis populations, a BNP threshold of 165 pg/mL has been proposed to identify pulmonary congestion confirmed by LUS.^20^In our cohort, a BNP cutoff value > 963.8 pg/mL predicted pulmonary edema on LUS with 70% sensitivity and 84% specificity.

Malnutrition in children with CHD is associated with adverse outcomes, including increased mortality, prolonged hospitalization, and longer intensive care unit stays in the preoperative period.^21 , 22^Approximately half of the patients in this study were underweight. Pulmonary edema detected by LUS was more frequent among underweight children and those with BMI-for-age below −2 SDs. Although previous studies have reported no significant impact of BMI on outcomes in CHD,^22^our findings suggest that nutritional status may influence the presence of pulmonary edema. Comprehensive assessment of anthropometric parameters may therefore contribute to improved risk stratification and prognosis prediction.

Several limitations should be considered. This was a retrospective, single-center study with a relatively small sample size and clinical heterogeneity regarding CHD type, age, medication use, and comorbidities. Imaging timing was not standardized, which may have influenced detection of pulmonary congestion. Although most patients were evaluated preoperatively, two postoperative cases were included. Despite these limitations, the study provides preliminary insights that may inform the design of future prospective investigations. Observer bias was minimized through blinded image interpretation.

## Conclusion

This study evaluated the role of LUS in detecting pulmonary edema in children with CHD. Abnormal LUS findings were significantly associated with underweight status, recurrent LRTIs, and increased BNP levels, which supports its value in identifying high-risk patients. Down syndrome was not associated with abnormal LUS findings, which suggests that it does not independently predispose patients to pulmonary congestion detectable by LUS.

Because of its bedside applicability and absence of radiation exposure, LUS appears to be a useful complementary modality to chest radiography in the preoperative assessment of pulmonary congestion in children with CHD. Further large, multicenter, prospective studies are needed to standardize LUS protocols, validate diagnostic performance, and determine its impact on clinical decision-making and patient outcomes across different CHD subtypes.
